# Undetected anteromedial coronoid fracture in elbow dislocation: A case report^☆^^☆☆^

**DOI:** 10.1016/j.tcr.2015.10.003

**Published:** 2015-11-14

**Authors:** R. Barco, D. Duran, S.A. Antuña

**Affiliations:** Shoulder and Elbow Unit, Orthopedic Surgery and Traumatology Department, La Paz Hospital, IDIPAZ, Madrid, Spain

**Keywords:** Anteromedial coronoid fracture, Coronoid fracture, Elbow dislocation, Posterolateral elbow instability, Lateral collateral ligament injury, Fixation

## Introduction

Posteromedial elbow instability has been described as an injury to the lateral ulnar collateral ligament (LUCL) and an anteromedial coronoid fracture, typically with absence of a radial head injury and mild incongruity of the elbow that can lead to a rapid onset of degenerative joint changes [1]. On the other hand, an acute posterolateral elbow instability pattern includes injury to the LUCL, anterior capsule and, less often, to the medial collateral ligament (MCL). [2] In a complex pattern, a fracture of the radial head and a fracture of the coronoid can be associated with increasing instability of the elbow [3]. Appropriate treatment protocols include repair of all the injured structures to restore elbow stability.

When assessing an elbow injury, we naturally interpret the available data to try to understand the mechanism of injury and it follows to assess the integrity of the typically associated injuries. However, the observation of a posterolateral elbow dislocation with absence of injury to the radial head may be interpreted as a simple elbow dislocation. The finding of a coronoid tip fracture may indicate a complex pattern of instability. The failure to adequately recognise the severity of coronoid fractures may lead to inadequate treatment.

We present a case that presented in the emergency room (ER) with a radiographic posterolateral elbow dislocation and absence of radial head injury with an unrecognised fracture of the anteromedial coronoid, typically associated with a posteromedial pattern of instability treated like a posterolateral simple elbow dislocation and a similar case with adequate recognition of the pattern and severity of the coronoid fracture.

## Materials and methods

**Fig. 1:**
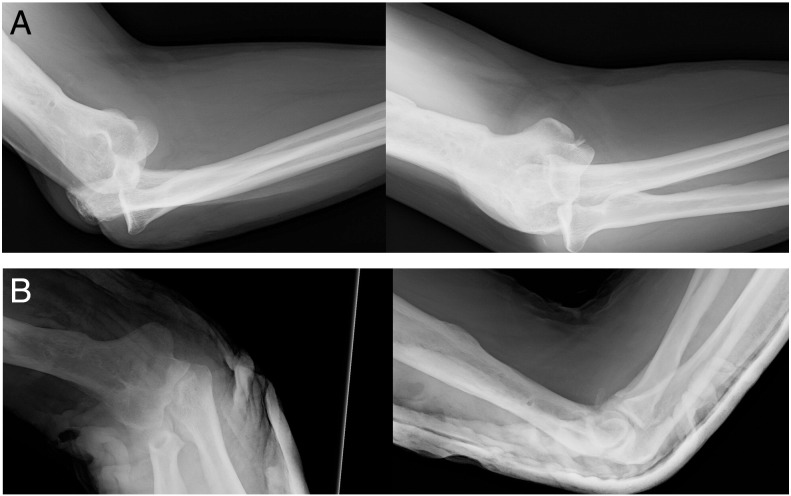
(A–B) Initial radiographic examination shows a posterolateral elbow dislocation and a fracture of the tip of the coronoid, typically associated with a posterolateral elbow pattern of instability (A). After reduction and splinting, the elbow is congruent with slight posterior sag of the radial head and a coronoid fragment is observed in front of the humerus. No injury to the anteromedial coronoid is apparent in these exams (B).

**Fig. 2:**
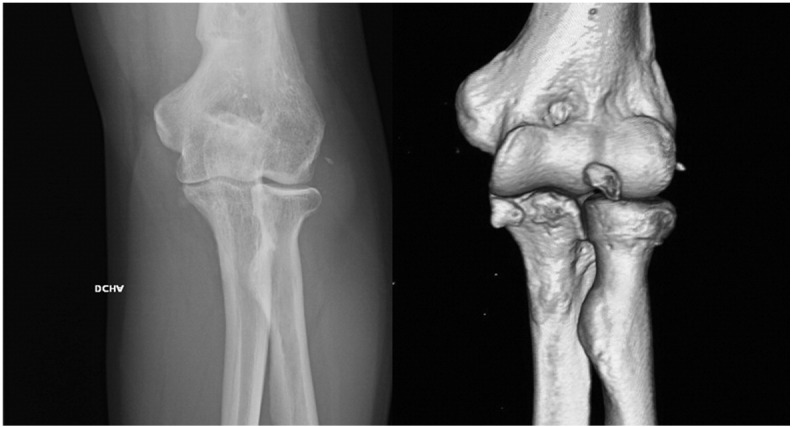
Six weeks radiographic examination of the right elbow shows medial joint space reduction so an anteromedial coronoid fracture was suspected and a CT scan was performed. A small anteromedial coronoid fracture is observed along with a fracture of the coronoid (O'Driscoll type II fracture). At this point in time, the reconstructive options are limited.

Case 1 A 60-year-old bricklayer presented in the ER with a radiographic posterolateral dislocation after a fall on the outstretched hand from his own height. After an initial x-ray exam, his elbow was reduced and placed in a splint at 90° of flexion after testing for stability in flexion and extension (Fig. 1.). He was immobilised for 2 weeks after which the cast was removed and the patient was started on active elbow motion exercises with avoidance of varus stress. At 6 weeks, the patient was visited and x-rays of the elbow were taken observing mild joint narrowing of the medial side of the elbow joint. The patient was referred to our clinic with a CT scan in which we observed an anteromedial fracture of the coronoid that was subtle on the initial radiographs (Fig. 2).

**Fig. 3:**
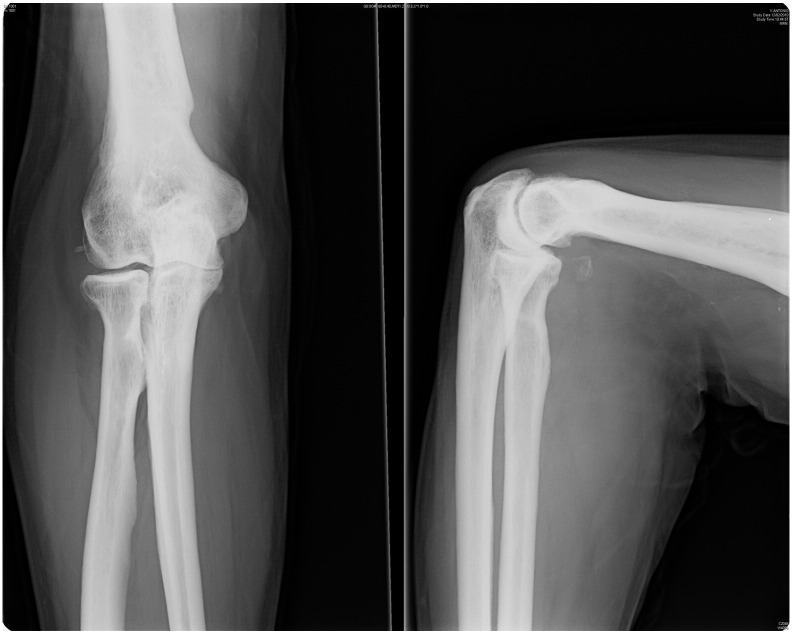
After 2 years of follow-up, the patient has good motion but has pain on the medial side of his elbow and has limited his activity.

At 2-month follow-up, the patient showed the decrease of the ulnohumeral joint with full range of motion and slight pain when loading the elbow (Fig. 3) that made him change his occupation to a less demanding job. The patient declined any surgical treatment and at 2 years shows medial-sided arthritis of the elbow with good elbow motion (20°–130°) and a stable joint.

**Fig. 4:**
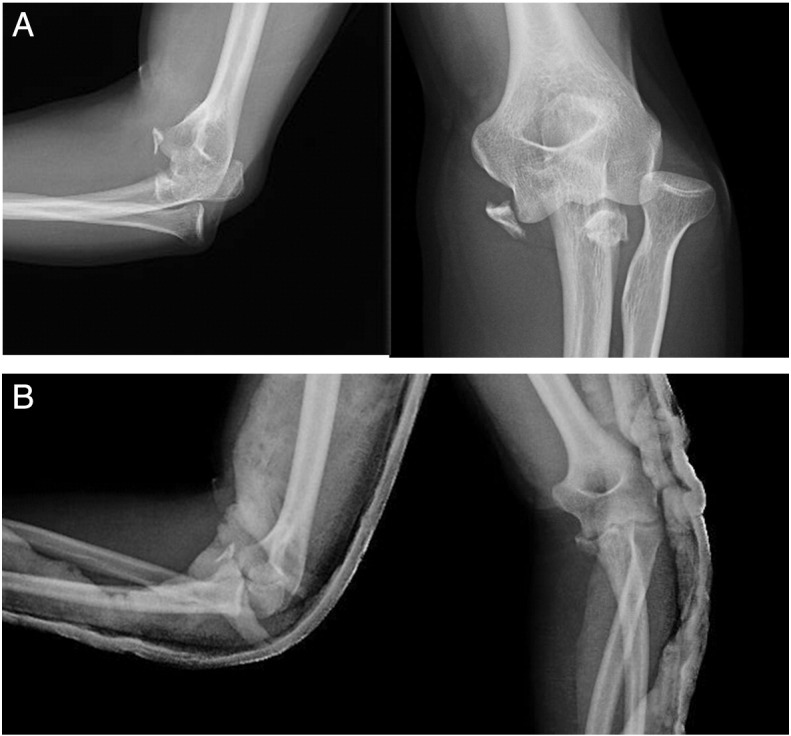
(A–B) Initial radiographic examination shows a posterolateral elbow dislocation and a fracture of the anteromedial coronoid (A). After reduction, a CT scan with 3D reconstruction was performed which better delineates the anteromedial fracture fragment and an associated anterolateral fracture fragment (B).

**Fig. 5:**
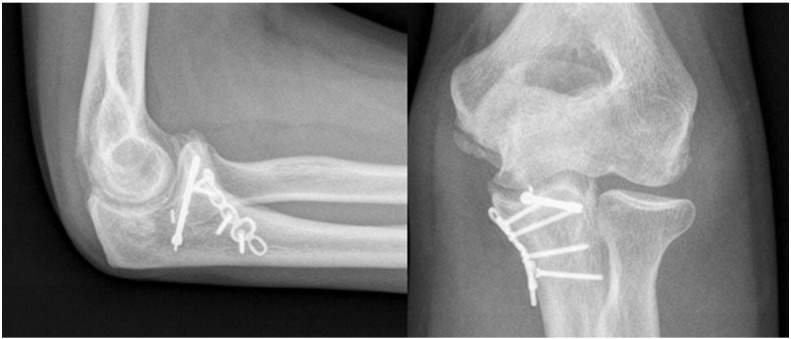
Surgery was performed through a posterior skin incision to reduce and fix the tip of the coronoid with a posterior-to-anterior screw, buttress plating of the anteromedial coronoid and repair of the LCL.

**Fig. 6:**
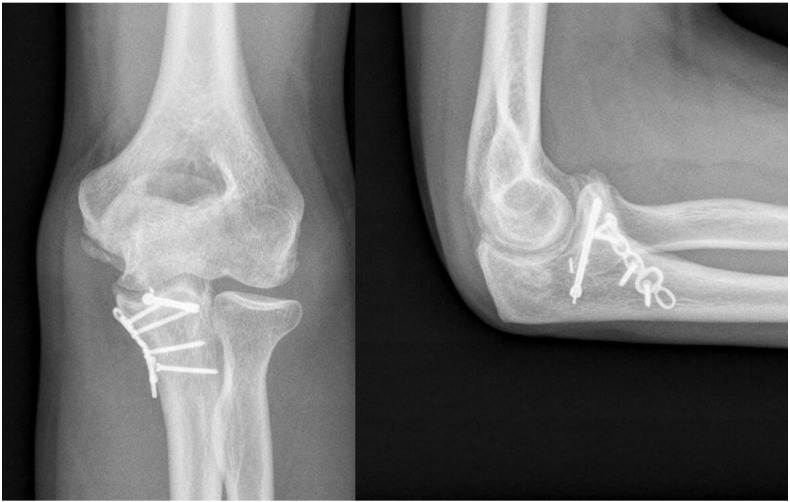
Postoperative radiographic exam at 2 years of follow-up shows good healing of the fracture and good alignment of the elbow. The patient has good motion and has returned to his pre-injury level of activity with no restrictions.

Case 2 A 30-year-old man presented in the ER referring a fall on his outstretched hand and exhibiting pain and clinical signs of elbow dislocation. On his x-ray examination, a posterolateral elbow dislocation with a readily recognisable fracture of the anteromedial coronoid is apparent (Fig. 4). The patient underwent elective surgery to stabilise the coronoid fracture with plate osteosynthesis through a medial approach between both heads of the flexor carpi ulnaris and repair of the LUCL with transosseus sutures and subcutaneous anterior transposition of the nerve (Fig. 5). The patient was placed in a splint for a week after which he began active elbow motion exercises with LUCL protection. At 2 years of follow-up, the patient has 5–135° of elbow motion with a stable elbow, good alignment and no pain (Fig. 6).

## Discussion

The observation of a posterolateral elbow dislocation on a roentgenographic exam may lead the treating physician to consider the existence of a posterolateral mechanism of injury with injury to the lateral ligamentous complex of the elbow and may direct the attention for associated injuries including radial head fractures and fractures of the tip of the coronoid [1]. However, the elbow can dislocate though a posteromedial varus mechanism and finally stay dislocated posterolaterally. As in the emergency room, the initial appearance of the elbow is in a posterolateral position, the initial assumption of this mechanism may lead to suboptimal treatment with failure to investigate further the pattern of the coronoid fracture.

O'Driscoll described the pathomechanics of posteromedial and posterolateral dislocation and it is believed that the injuries to the different ligamentous structures are sequential [1]. A posterolateral pattern of instability can be associated with radial head fractures and coronoid fractures and have less favourable outcomes with an increased rate of complications [1], [4]. A surgical protocol including repair of the coronoid, the radial head, and the LUCL has improved the results of this complex injury [5]. Medial compression fractures of the coronoid have been described and are recognised as difficult fractures to diagnose with dire consequences for the joint if associated with joint incongruity [6]. The importance of the coronoid as a stabiliser for varus and posteromedial ulnar translation in the elbow has been recognised and introduced in O'Driscoll's classification of coronoid fractures [1], [7], [8]. Posteromedial instability has been described as an injury to the lateral ulnar collateral ligament (LUCL) and associated anteromedial coronoid compression fracture, typically with absence of a radial head injury and mild incongruity of the elbow joint. The fracture line usually follows a more sagittal orientation than other types of coronoid fractures [3]. In cases of undisplaced fragments, good results may be achieved with conservative management, but if associated with joint incongruity, articular degeneration occurs very fast, so it is recommended to achieve an anatomical reduction and stable fixation of the fracture with associated LUCL repair [9]. In cases of comminution or a tenuous repair, the use of an external fixator can be useful to protect the elbow while the fracture heals. Injuries to the anteromedial coronoid can be very subtle and may be easily missed on an x-ray exam. More frequently, anteromedial fractures are associated with tip fractures of the coronoid (O'Driscoll type II), so the finding of a tip fracture on a lateral x-ray should prompt a higher scrutiny for detection of an anteromedial fracture with the use of CT scan, preferably with 3D image reconstruction. It is the case that both patients had an associated fracture of the tip of the coronoid with the patient achieving an optimal result when the anteromedial fragment was diagnosed and adequately treated. Failure to recognise this pattern of injury may lead to suboptimal results.

The detection of associated injuries is critical to identify the type of elbow instability and to apply the correct treatment. The majority of posterolateral elbow dislocations presenting in the ER are due to a posterolateral pattern of injury, but these cases show that some of these may be produced following a posteromedial pattern of instability. Obviously, the detection of the anteromedial fragment is obvious in case 2 and led to proper treatment and a good outcome. In case number 1, failure to detect an anteromedial coronoid fracture led to the assumption that it was a posterolateral elbow dislocation with a small coronoid tip fracture and led to suboptimal treatment.

Simple elbow dislocations typically obtain good outcomes after reduction and a short period of immobilisation [1]. The results of complex elbow dislocations are less favourable and depend on the identification and proper treatment of the associated injuries [8]. The detection of a small anteromedial fragment in a case with an episode of documented posterolateral instability may be difficult unless a CT exam is performed and may be associated with a poor outcome [10]. Doornberg et al. recognised this pattern as a terrible-triad dislocation with a fracture of the anteromedial facet of the coronoid process with an associated fracture of the coronoid tip and a marginal fracture of the radial head on CT exam, although the initial radiograph did not show a fracture of the radial head. Interestingly, they correlate terrible-triad injuries with a posterolateral mechanism of injury and fractures of the anteromedial facet with a posteromedial pattern of injury [3].

Evidence of a posterolateral elbow dislocation on a presenting x-ray exam in the ER does not equate to the assumption of a posterolateral elbow mechanism. In posterolateral elbow dislocations, the search for associated injuries should include the anteromedial coronoid fractures, specifically in cases with absence of fracture of the radial head or with an associated tip fracture of the coronoid and appropriate management should be instituted to avoid early degenerative changes of the joint.

## Conclusion

Posterolateral elbow dislocations are the most frequent type of elbow dislocation but may follow a posteromedial instability pattern. The failure to detect and treat correctly an associated anteromedial coronoid fracture may compromise the outcome. A high index of suspicion for the presence of an associated anteromedial coronoid fracture may be established when a posterolateral elbow dislocation presents with absence of a radial head fracture and appropriate imaging techniques utilised for its detection.
